# Improving Autonomous Robotic Navigation Using Imitation Learning

**DOI:** 10.3389/frobt.2021.627730

**Published:** 2021-06-01

**Authors:** Brian Cèsar-Tondreau, Garrett Warnell, Ethan Stump, Kevin Kochersberger, Nicholas R. Waytowich

**Affiliations:** ^1^ Unmanned Systems Laboratory, Mechanical Engineering, Virginia Polytechnic Institute and State University, Blacksburg, VA, United States; ^2^ Army Research Laboratory, Adelphi, MD, United States

**Keywords:** autonomous navigation, learning from demonstration, imitation learning, human in the loop, robot learning and behavior adaptation

## Abstract

Autonomous navigation to a specified waypoint is traditionally accomplished with a layered stack of global path planning and local motion planning modules that generate feasible and obstacle-free trajectories. While these modules can be modified to meet task-specific constraints and user preferences, current modification procedures require substantial effort on the part of an expert roboticist with a great deal of technical training. In this paper, we simplify this process by inserting a Machine Learning module between the global path planning and local motion planning modules of an off-the shelf navigation stack. This model can be trained with human demonstrations of the preferred navigation behavior, using a training procedure based on Behavioral Cloning, allowing for an intuitive modification of the navigation policy by non-technical users to suit task-specific constraints. We find that our approach can successfully adapt a robot’s navigation behavior to become more like that of a demonstrator. Moreover, for a fixed amount of demonstration data, we find that the proposed technique compares favorably to recent baselines with respect to both navigation success rate and trajectory similarity to the demonstrator.

## 1 Introduction

Decades of work in autonomous robot navigation have resulted in a well-studied and successful framework for safe and efficient traversal in known and unknown environments. Given an accurate environment map, autonomous navigation can be accomplished with a combination of global path planning (Kavraki et al., 1996; LaValle and Kuffner Jr, 2001) and local motion control (Petrovic, 2018; Thrun, 1998). Valid trajectories are typically computed by optimizing a pre-specified cost function that measures factors such as path length, chance of collision, and execution time (Petrovic, 2018).

However, adapting this standard framework to exhibit unanticipated but necessary navigation behaviors can be challenging. The need for such adaptation arises because what constitutes a valid trajectory can vary across users, tasks, and the specific environments in which the agent is deployed. For example, in situations such as disaster robotics (Doroodgar et al., 2014; Liu et al., 2012), the ability to perform on-the-fly adaption of navigation behaviors in light of new information in dynamic environmental conditions (e.g., decreased traversability of roads due to partial flooding), may prove critical to the success of the mission. Explicitly modifying the navigation cost function to induce valid trajectories may be possible, but doing so accurately and quickly currently requires substantial effort from an expert roboticist with a great deal of technical training, if it can be done at all. Therefore, we seek alternate methods for incorporating user preferences into autonomous navigation systems that do not involve explicitly re-writing computer code or specifying cost functions and are accessible to users without training in robotics. Combining autonomous navigation with Machine Learning is a promising approach by which such alternate methods might be developed. In particular, Learning from Demonstration (LfD) (Argall et al., 2009)—in which a machine learner attempts to imitate the behavior of a demonstrator—is directly applicable to the problem of autonomous navigation since many humans can successfully provide demonstrations by tele-operating the platform. Unfortunately, precisely how LfD approaches should be applied to the problem of autonomous navigation still remains unclear. Existing approaches based on Inverse Reinforcement Learning (Wigness et al., 2018) may incur large training costs, and approaches based on Behavioral Cloning (BC) (Bojarski et al., 2016) are limited by common issues such as data distribution shift (Ross et al., 2010) and lack of generalization. Furthermore, many existing approaches incur a prohibitively large human cost in terms of amount of demonstration data required (Neu and Szepesvári, 2012; Pfeiffer et al., 2018).

In this paper, we propose a new framework for combining autonomous navigation and LfD to overcome these limitations. The key idea is to combat the costs inherent in pure Machine Learning approaches by leveraging an appropriate amount of the existing machinery of planning and control. This idea has been successfully used in other contexts in the form of a learned navigation system that can propose intermediate waypoints (Bansal et al., 2019) or approximate planner costs that have no easy calculation (Stein et al., 2018). Such hybrid approaches leverage the results and guarantees of decades of work in optimal control and planning but take advantage of specific locations where heuristics can speed up or shape the results.

Our proposed approach adopts such a hybrid approach in the context of LfD in two novel ways: 1) by integrating BC directly into an off-the-shelf navigation stack through a module we insert between the existing global path planner and the local motion planner, and 2) by using a training paradigm that uses demonstrations from both humans and a classical navigation stack to increase the overall system success rate. With respect to (1) in particular, the inserted module takes input from both the platform’s on-board sensors and the global path planner to produce intermediate goals for the local planner. Unlike traditional systems in which these intermediate goals are specified by the global planner, the goals produced by our module are the output of a learned function that is trained to emulate navigation behavior as demonstrated through tele-operation by a human. We study the proposed method experimentally in a simulated environment. Specifically, for a fixed amount of demonstration data, we seek to characterize the efficacy of our approach in terms of both the similarity of the produced navigation trajectories to that of the demonstrator as well as the overall navigation task success rate. We evaluate the performance of our proposed method in two simulation environments: 1) a simple proof of concept environment simulated in Gazebo (Koenig and Howard, 2004), and 2) a more realistic and complex environment simulated in Unity (Unity Technologies). We compare our technique to recently-proposed baselines that also propose to incorporate LfD with autonomous navigation, and we find that the proposed technique excels in performance.

## 2 Related Work

In this section, we discuss classical approaches for robotic navigation, followed by a brief exploration of Machine Learning from Demonstration, then conclude this section by highlighting recent hybrid approaches that try to combine the two.

Classical robot navigation is accomplished with a hierarchical suite of navigation software. Specifically, a global path planning module that generates optimal paths given an occupancy map, and local planner that executes feasible control signals that adhere to the robot’s kinematic constraints and a mathematically defined objective function (Kalakrishnan et al., 2011; Kavraki et al., 1996; Laumond et al., 1994; LaValle and Kuffner Jr, 2001). While this setup can reliably generate geometrically optimal trajectories and motions over long distances, navigation trajectories/behaviors that are optimal with respect to user preferences or constraints are not easily defined mathematically. Thus, necessitating inclusion of Machine Learning approaches to traditional path planning.

Recent interest in improving upon traditional robot navigation have been geared towards leveraging Machine Learning techniques such as Learning from Demonstration (LfD) to help facilitate robot navigation. Learning from Demonstrations can be decomposed into two general areas based on the approach: Behavioral Cloning (BC), an application of Supervised Learning where a mapping from observations to actions is learned (Ross et al., 2010) and Inverse Reinforcement Learning (IRL), which generates a reward function that explains the demonstrated behavior (Abbeel et al., 2004; Ziebart et al., 2008). Some approaches attempt to use Machine Learning in isolation for handling robot navigation. For example, Chiang et al. frame the navigation task as an end-to-end learning problem to predict continuous controls (e.g., steering angle, translational and rotational velocities) directly from raw state observations (Lewis Chiang et al., 2018).

Other recent works attempt to combine Machine Learning and classical navigation as an intuitive means to quickly model preferences for a navigation task with the help of a human teacher. Wigness et al. (2018) and Siva et al. (2019), combine IRL and visual feature extraction to train navigation policies in unstructured terrains from human demonstrations. However, methods for IRL involve an iterative constrained search through the space of reward functions, leading to a disproportionate growth in solution complexity with the problem size. Müller et al. (2018) aim to transfer driving policies from simulation to physical platforms and achieve this by learning policies that output a series of waypoints to be followed by a lower-level planning and control system. They focused on a general navigation task rather than consideration of how this setup might be used as a mechanism for Imitation Learning. Gao et al.’s intention-net (Gao et al., 2017), and Pokle et al.’s work (Pokle et al., 2019) are similar to our own approach as they utilize the global planner provide the general direction that a robot should travel to reach a desired destination in a known environment. These methods attempt to address the shortcomings of classical navigation with Machine Learning at the cost of high training time, complexity, and data efficiency by training models to replace the functionality of proven low level controllers used in classical navigation techniques. Instead of replacing the local controller, our approach integrates a trained policy as an intermediate module between the path planner and local controller modules found in any off-the shelf navigation software. This effectively combines an intuitive interface for a human user to adapt a proven, but inflexible, navigation control scheme with their own preferences and behavior on how the navigation problem should be solved.

Our key contributions are twofold. First, we introduce a novel and efficient training scheme for using LfD in autonomous navigation that contrasts with current-state of the art approaches. Second, we provide empirical evidence that our new that the proposed method can achieve high path completion rates in both familiar training and novel testing environments while adhering to an implicitly defined navigation rule embedded in human demonstration.

## 3 Approach

**FIGURE 1:**
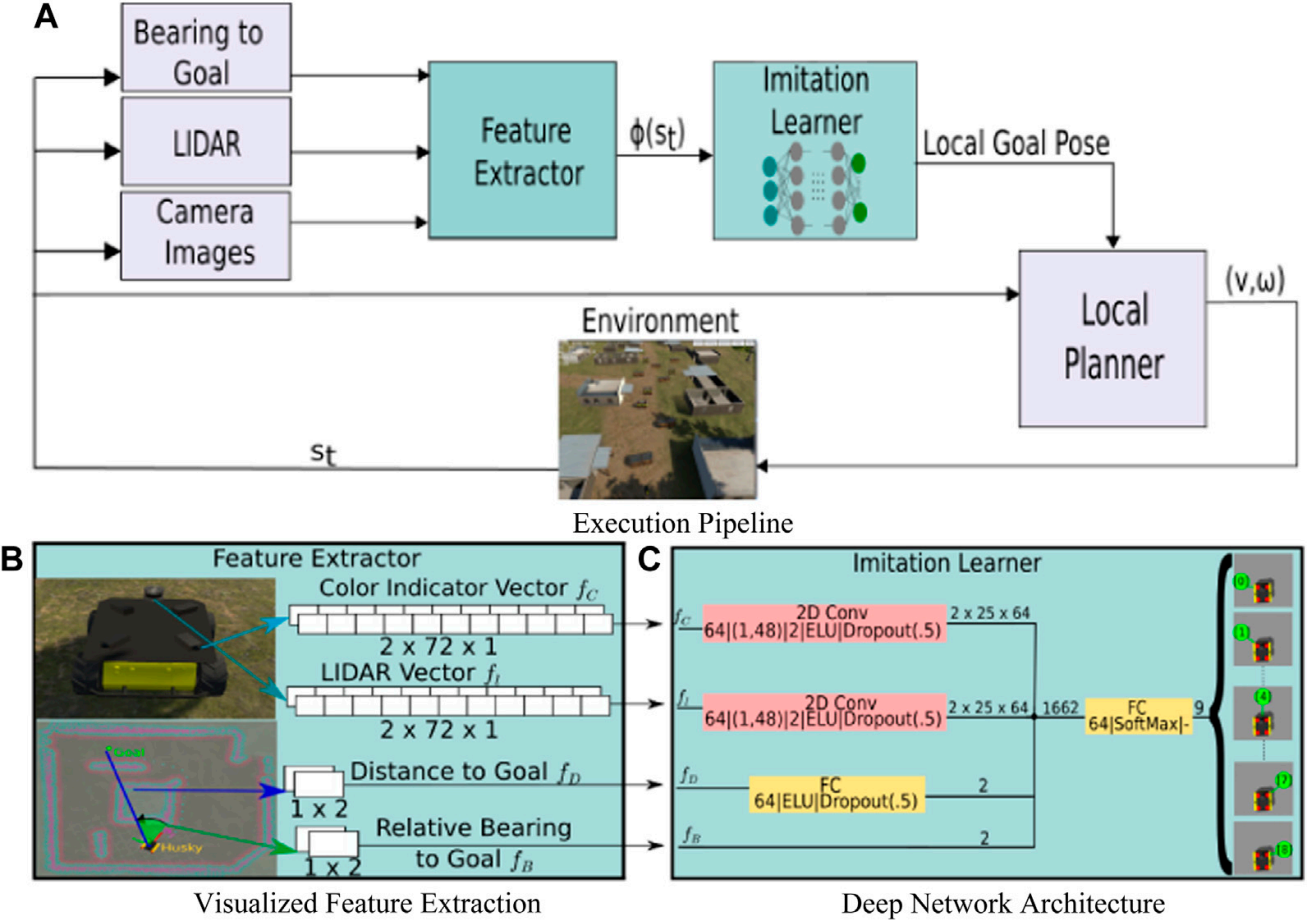
**(A)** Proposed execution pipeline for improved autonomous navigation: a neural network is inserted before the local planner in the default ROS navigation stack. This network is trained using BC from human demonstrations to map current state and task information to goals for the local planner. **(B)** Visualization of image features, lidar data, distance to goal, and relative bearing to goal features are collected and transformed to low dimensional inputs to the policy model. **(C)** The Imitation Learning module that maps the observations of the current state, pre-processed in the feature extraction step, to a local goal for the local planner.

Our proposed approach integrates a behavior cloning model directly into an off-the-shelf navigation stack as a means to enable adaptation *via* human-demonstrated navigation behaviors. Figure 1 shows a system diagram of the proposed architecture, wherein our behavior cloning model takes as input the current bearing to the global goal and a windowed sequence of sensory and state estimation information, and provides as output a local goal for the local planner. In this section we provide a general overview of the proposed system architecture, followed by our model training and demonstration collection procedure.

### 3.1 State Space and Feature Extraction

We modify a traditional layered navigation stack of global and local path planning modules by inserting between them a new Machine Learning module. This module accepts as input both platform sensor data as well as features related to the global planner, and outputs goals for the local planner. Below, we describe the state and action spaces for our Machine Learning module in more detail.

The feature extraction and compilation process we use is detailed in Figure 1B. The features include perceptual observations of the current state (i.e., visual cues extracted from images or spatial information from a planar lidar) and task information in the form of the current distance and relative bearing to the navigation goal. As a means by which to supply the imitation learner with historical context, we also employ a temporal stacking technique for each of the above features. In our work, as shown in Figure 1B, we stack m=2 sequential (current and previous time instant) features. For the perceptual observations $f_{c}$ and $f_{l}$, this amounts to moving from size 72×1 each to 2×72×1 each. For the task features, $f_{B}$ goes from 1×20 to 1×40 and $f_{D}$ from a scalar to 1×2. The state space, $s_{t}\in S$, is a low dimensional representation of the free space in the environment, and is comprised of the following features:

Lidar features: The lidar feature vector, $f_{l}$, consists of *n* evenly sampled laser range points from a 360° planar lidar. Here, n=72, which corresponds to a lidar measurement sampled every 5° (see Figure 1B).
Visual features: The visual feature vector, $f_{C}$, is processed from a panoramic RGB image of the robot’s surroundings that underwent pixel level semantic labelling of the objects in its visual frame. Specifically, the panoramic RGB image can be generated by a camera with a 360° horizontal field of view, or several cameras arranged about the robot chassis to produce a similar image. From the RGB image(s), we generate a pixel level semantic image, labeling each object type (e.g., crate, building, gravel, etc) in the image frame with a unique color (see Figure 3F). While our simulator directly generates these semantically labeled images, in real world environments, one can acquire similar ones using deep networks for classification and image segmentation such as Mask RCNN (He et al., 2017) or DeepLabv3 (Chen et al., 2017). Each element in $f_{C}$ indicates the presence of an object of interest (such as color, texture, or pattern) within a segment of the camera’s horizontal field of view (HFOV) (see Figures 1B,3E).
Goal bearing feature: The goal bearing feature, $f_{B}$, provides our network with the navigation goal location’s relative bearing to the robot at time *t*. It serves to orient the model’s predicted actions in the direction of the goal even when maneuvers to avoid obstacles or adhere to human navigation preferences leave the robot facing opposite to the goal location (see Figure 1B).
Distance feature: $f_{D}$ is the euclidean distance between the robot and the desired goal location at time *t*. We use distance to the goal to avoid training the BC model to navigate to specific goal locations used in training. This allows the policy to generalize its behavior to goal locations not seen during training (see Figure 1B).

### 3.2 Action Space

**FIGURE 2:**
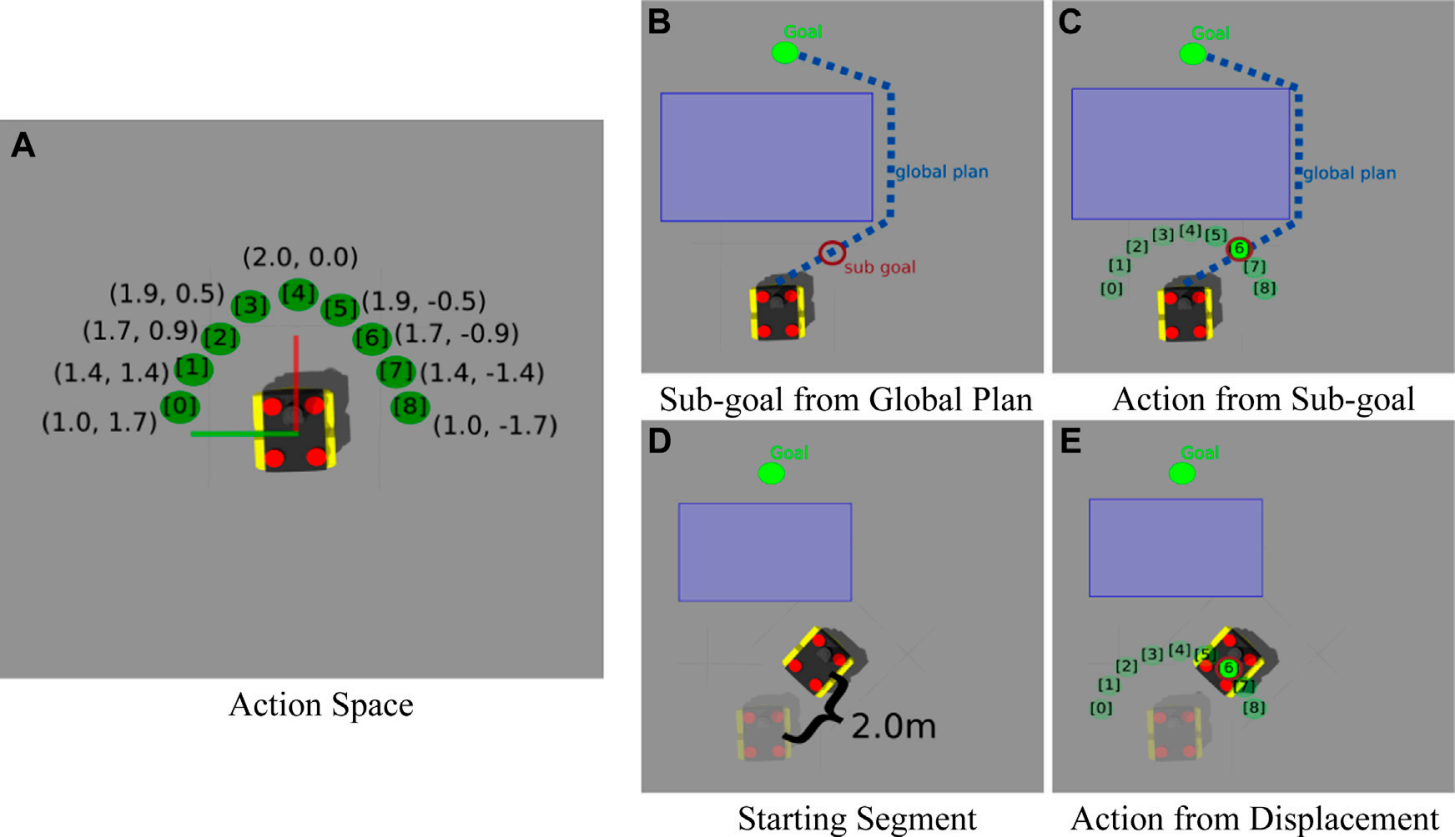
**(A)** displays each local waypoint $a_{l}$ comprising our action space A and their respective coordinates in the robot’s reference frame. **(B)** and **(C)** show how we extract the actions demonstrated by the navigation stack as described in Section 3.4.1. In **(B)** a sub-goal in front of the robot is selected from the global plan from which **(C)** the closest action, $a_{l}$, in the action space, is inferred. Panel **(D)** and **(E)** show how we extract the actions demonstrated by human or expert user described in Section 3.4.2. **(D)** shows the starting segment when the robot has been moved to a new pose approximately 2.0 m away from its pose in the starting segment, after the current state, $s_{t}$ was recorded. Finally, in **(E)**, the action, $a_{l}$ is selected as the one that yields most similar displacement to that observed in **(D)**.

As discussed in Section 2, most methods combining Machine Learning with classical navigation, train a model that directly predicts low level control signals from raw sensory data, effectively replacing the local planner. Our model, however, takes advantage of the local motion planning module included in the ROS navigation stack. To do so, our model predicts one of L=9 class labels, each of which corresponds to a 2D waypoint $A=\left\{a_{l}|l=0,\ldots ,L-1\right\}$. Each waypoint was sampled at predefined angular intervals along the arc of a semicircle in front the robot in its coordinate frame of reference (see Figure 2A). Given the current observation of the state, $s_{t}$, our BC model outputs *L* scores—one for each predefined waypoint—and the waypoint with the highest score is then passed to the local planner. This leaves the computation of valid low-level control signals to the local planner which is specifically designed to consider the kinematic constraints of the robot platform in its plan. Over the course of the model’s execution, the model’s predicted waypoints should result in a path that adheres to the demonstrated user preferences.

### 3.3 Network Architecture

Our model takes in four separate inputs: state features $f_{l}$ and $f_{C}$ respectively, the relative bearing to goal $f_{B}$, and the robots distance to the goal location $f_{D}$. $f_{l}$ and $f_{C}$ represent relevant environmental features observed about the robot. As such, the elements in $f_{l}$ and $f_{C}$ are spatially correlated which we leverage by performing a single channel convolution across their respective spatial dimensions with 64 hidden units. Each of the resulting feature maps are passed through a max pooling operation, followed by a dropout regularization of 50%, and finally flattened to a vector that encodes the robots proximity to all obstacles in the immediate vicinity and the presence of visually interesting objects of interest in its field of view. The feature vectors resulting from the above operations and the unperturbed relative bearing to goal $f_{B}$ and distance features $f_{D}$ are concatenated before finally being passed through a fully connected output layer with SoftMax activation, and nine hidden units matching the discrete action labels comprising our action space. Finally, the model is optimized using categorical cross entropy loss.

While we did not perform an exhaustive hyperparameter search for the neural network model, we were able to find a setting that worked well for our purposes. Given the small number of examples we use during training, adding a dropout layer at 50% was necessary to prevent over-fitting. The 64 hidden units in the convolutional and dense layers were enough to provide a large enough number of parameters for the model to learn new behaviors from additional demonstrations without experiencing catastrophic forgetting. We leave further search and refinement of the neural network architecture to future work.

### 3.4 Training Procedure

Our proposed method uses a two phase training procedure: a pre-training phase followed by a human update phase. First, in the pre-training phase (see Section 3.4.1), an initial navigation policy, $\pi_{\theta_{0}}$, is trained using behavioral cloning on a dataset of demonstration trajectories, $D_{nav}=\left\{\tau_{0},\tau_{1}\ldots .\right\}$, where each trajectory, τ, is comprised of a sequence of state action pairs $\left[\left(s_{0},a_{0}\right),\left(s_{1},a_{1}\right),\ldots \right]$ generated by the ROS move_base navigation stack. Second, in the human update training phase (see Section 3.4.2), starting from $\pi_{\theta_{0}}$, we again use behavioral cloning to find our navigation policy $\pi_{\theta^{\text{*}}}$ using demonstrations $D_{expert}=\left\{\tau_{0},\tau_{1}\ldots .\right\}$ from a human tele-operating the robot between a predefined set of start and end locations within the same simulated training environment in a way that adheres to an implicit navigation rule. It should be noted that, in order to reduce effort on the part of a human expert to generate demonstration trajectories, $\left|D_{nav}|\gg |D_{expert}\right|$.

This two phase training procedure allows the user to focus on providing good demonstrations of their navigation preferences in the second phase, that will be used to modify a “vanilla” navigation policy trained in the first phase. The total number of training demonstrations required by our proposed method is far fewer than other works that utilize the classical navigation modules in their training procedure (Gao et al., 2017; Pokle et al., 2019). This is because neither one of the training procedures used in our method is trying to create a policy that completely replaces classical navigation modules. Since we still employ these modules at test time, the trained model need not account for kinematic constraints or recovery behaviors.

#### 3.4.1 Pre-Training Procedure

We train the initial policy network, $\pi_{\theta_{0}}$, with demonstrations $D_{nav}$ only (we describe the collection procedure in Section 4.2) until either convergence or for a fixed number of epochs. We use a categorical cross entropy loss function between the action in the demonstration, $a_{i}$, and the predicted action label from the network, $\hat{a_{i}}$, i.e.,

$$Loss=-\sum_{i=0}^{L-1}a_{i}log\left(\hat{a_{i}}\right).$$ (1)

$D_{nav}$ is obtained by recording the state-action sequence (i.e., τ) observed by the robot being driven by a modified off-the-shelf navigation system tasked to navigate between randomly-generated start and end locations in the simulated training environment (see Algorithm 1 line 2). The demonstrations are obtained by first observing and recording the robot’s state, $s_{t}$, and then selecting the closest sub-goal from the navigation system’s global plan that is at least 2.0 m in front of the robot (see Figure 2B). We then pick the action $a_{t}$ in our action space that is closest to that sub-goal, and task the navigation system’s local planner to move the robot to the local waypoint corresponding to $a_{t}$ (see Figure 2C). This process is repeated until the robot reaches the goal and results in a demonstration trajectory τ with state-action pairs that align with the state and action space used by the learner. In practice, we stack the *m* most recent states and use this stack as the input to our learner.

**Table:** 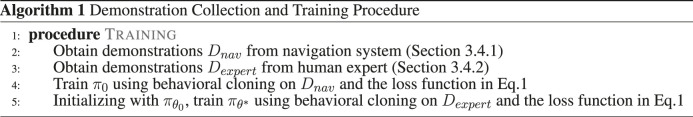

#### 3.4.2 Training With Human Updates

After the initial navigation policy, $\pi_{\theta_{0}}$, is trained, we update it with several human demonstrations, where the user tele-operates the robot in a way that adheres to one or more semantic rules or preferences (e.g., navigate a wide berth about objects with specific features or always keep said objects to the right of the robot). Demonstrations comprising $D_{expert}$ were recorded by dividing the tele-operation history into distinct segments of 2.0 m displacement (see Algorithm 1 line 3). The state of the robot at the start of each segment was recorded as $s_{t}$ (see Figure 2D), and the action $a_{l}$ was selected as the one that would yield a displacement most similar to that observed in the demonstration segment (see Figure 2E). These demonstrations are then used to introduce human demonstrated navigation preferences or task-specific behaviors by initializing the navigation policy $\pi_{\theta^{\text{*}}}$ with $\pi_{\theta_{0}}$ and then performing supervised learning using $D_{expert}$ and the loss function shown in Eq. 1.

## 4 Experiments

We perform simulation experiments with the goal of characterizing the efficacy of our approach in terms of both the similarity of the produced navigation trajectories to that of a demonstrator and the overall navigation task success rate. Specifically, the goal is to successfully navigate between a start and goal location while adhering to an implicit navigation style, to navigate a wide berth around any objects with a specific semantic label encountered, that the behavior cloning module learned from human demonstrations as a result of Section 3.4.2. We compare our approach to recent baselines from Pfieffer et al. (2018) andBojarski et al. (2016) and find that our proposed method outperforms each with respect to both similarity to human demonstrations as well as navigation success rate. We do not provide an empirical analysis comparing our proposed method and baseline performances to traditional navigation stack behaviors. We expect that traditional methods would have a near-perfect navigation completion success ratio. However, they do not have the capacity to learn from demonstrations, and therefore would not be able to modify their behavior to incorporate human navigation preferences.

### 4.1 Experimental Setup

Experiments were performed using a 0.990×0.670×0.390 m simulated Clearpath Husky. This Husky is a ground vehicle with 4-wheel differential drive kinematics, a 360° planar lidar, and four on-board cameras, each with a 120° horizontal field of view. Each camera is mounted at one of the four corners on the Husky’s chassis facing outwards, and oriented such that they collectively provide a 360° visual coverage of the Husky’s surroundings.

**FIGURE 3:**
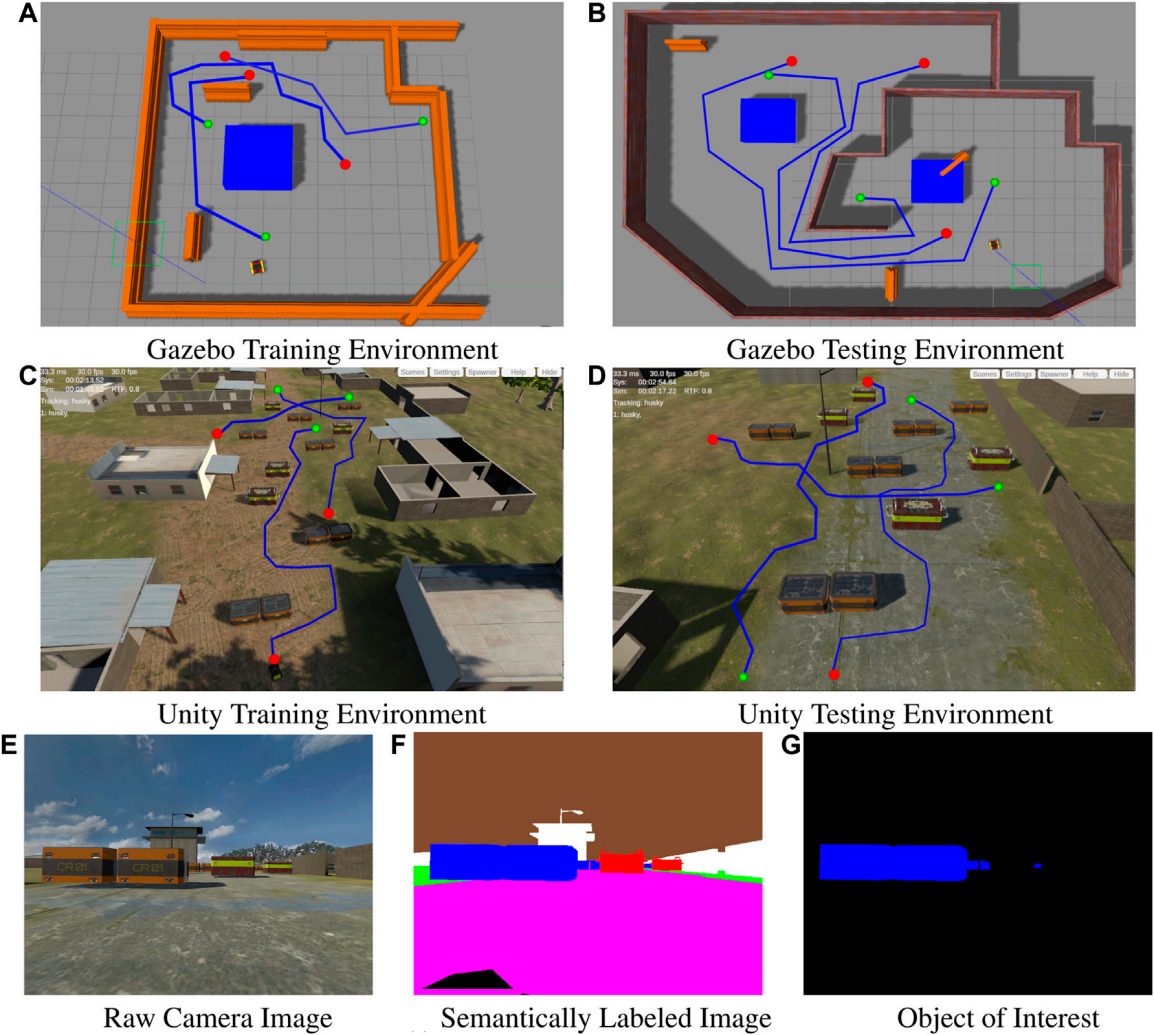
The top row shows a top down view of the of the **(A)** training and **(B)** testing environments simulated in Gazebo. The second row shows a top down view of the **(C)** training and **(D)** testing environments simulated in Unity. Images **(A)** through **(D)** display the exemplar paths generated by the human demonstrator starting from the red dot to the goal location at the green dot. The bottom row of images displays the raw camera image **(E)**, the semantically segmented version of the raw image **(F)** and the object of interest extracted from the semantic image **(G)** used to generate the visual feature vector *f*_*C*_.

Experiments were conducted using two different simulators. The first set of experiments uses the Gazebo simulator and focuses on two simplistic environments: a 10×12 m *training environment* (Figure 3A) and a 21×17 m *testing environment* (Figure 3B). The obstacles within each environment included a large blue box, brick walls, and orange Jersey barriers. Our second set of experiments was performed in a semi-urban environment created using the Unity simulator. Here again we conducted experiments in a 62×31 m *training environment* (Figure 3C) and a 48×21 m *testing environment* (Figure 3D). The obstacles within these environments include two kinds of crates, several buildings, and lamp posts. The temporal stack is initialized with zero vectors of the same dimensions as our model’s input, $s_{t}$. The state observed at time *t*, $s_{t}$, is pushed into the temporal stack and automatically removes the oldest element, $s_{t-m}$. The temporal stack is passed as input to the BC model, $\pi_{\theta^{\prime }}$, from which it predicts the class label, *l*, of the local waypoint, $a_{l}$, that is executed by the local planner. All training demonstrations took place in the respective simulator’s training environment, while evaluation of each resulting model took place in both the training and testing environments using navigation tasks (i.e., pairs of starting and ending points) that were not explicitly part of the training set. In particular, we considered six different navigation tasks for evaluation: three tasks in the training environment and three in the testing environment (visualized by the red blue and green paths in Figures 3A–D). For each model and navigation task evaluated, we performed 50 trials, i.e., we recorded the path taken by each model during the trial and whether the system made it to the destination or not. We consider trajectories that reach within 0.8 m of the goal location successful, and those that collide with an obstacle or exceed a 100 timesteps as failures.

The experimental scenario conducted in the Gazebo-based environments as shown in Figures 3A,B, are two enclosed rooms with large blocks acting as obstacles in an area between the robot’s starting location and the goal location. The large blocks are assigned a blue semantic label. Similarly, the experimental scenario conducted in the Unity-based environments, has several crates acting as obstacles in an area between the robot’s starting location and the goal location (as shown in Figures 3A,B). Some of the crates are semantically labeled i.e the orange crates and the red and yellow crates in Figure 3E being assigned the blue and red semantic labels respectively in Figure 3F. In both scenarios, the obstacles with the blue semantic labels represent the obstacle that should be traversed in the style of the human demonstrator.

The comparison objective is to empirically measure the similarities of the trajectories generated by a robot, navigating under the control of a model trained using our proposed method, and the experimental baselines, to the exemplar trajectories demonstrated by a human user through both the training and testing environments (i.e., the blue paths shown in Figures 3A–D). In essence, we should see that a robot, controlled by a model trained using our approach, successfully travels from start to goal, while maintaining a wide distance from semantically labeled obstacles it encounters.

### 4.2 Demonstration Data Collection

As described in Section 3.4, we first trained an initial behavior cloning model, $\pi_{\theta_{0}}$, to mimic the lowest cost to goal behavior of the existing move_base navigation software in the ROS navigation stack. $\pi_{\theta_{0}}$ was trained for 90 epochs, with a batch training size of nine examples per batch. To do this, we used move_base to generate the 200 demonstrations of the robot autonomously navigating from a randomly-sampled start location to a randomly-sampled goal location, within each of the *training environments* respectively, and record them into $D_{nav}$.

To obtain $D_{expert}$, the author provided demonstrations completing three different navigation tasks, recording four trajectories per task within each of the *training environments* respectively (i.e., 12 trajectories in total for each *training environment*). The author generated these demonstrations by tele-operating the robot in a way that accomplishes each task while adhering to the following rule: navigate a wide berth around any obstacles encountered that have the semantic label. Afterwords, we update the initial policy $\pi_{\theta_{0}}$ with $D_{expert}$, to obtain $\pi_{\theta^{\text{*}}}$. During this step, one must pay close attention to the number of training epochs used when updating to avoid catastrophic forgetting of the initial policy behaviors when introducing the human demonstration behaviors. The amount of training epochs used when updating $\pi_{\theta_{0}}$, was experimentally determined to be around 50 epochs, with a batch training size of nine examples per batch. The author also provided 12 additional task demonstrations (four trajectories per task) that are omitted from the training dataset. Three of which were novel tasks within the Gazebo *training environment* (see Figure 3A), three in the Gazebo *testing environment* (see Figure 3B), three in the Unity *training environment* (see Figure 3C) and in the Unity *testing environment* (see Figure 3D). These demonstrations are used to evaluate the performance of the models trained using our proposed method and baseline methods.

#### 4.2.1 Data Augmentation

To combat distributional shift in BC, we implemented a data augmentation method inspired by the work of Bojarski et al. (2016). For a given state-action pair in the demonstration, we produce two additional pairs by synthetically applying a rotational transform to the $f_{l},f_{C},f_{B},f_{D}$ features comprising $s_{t}$ such that they represent the features that would have been observed if the robot was oriented ± 45° from its current pose, and a corrective action that would be necessary to bring the robot back to a familiar state along the demonstrated trajectory under the associated transformation. This augmentation step effectively triples the amount of training data obtained from each demonstration *without* requiring any additional sensors or demonstrator time. We will henceforth refer to the original state-action pairs as coming from the *main frame* of reference and distinguish them from these augmented state-action pairs coming from the *corrective frame* of reference. We also have a specific procedure for temporal stacking corrective frame data before training commences the latest state observation from one of the corrective frames is stacked on top of the m−1 main frame observations that precede it. This assumes that the corrective frame states will temporally follow the main frame state(s), providing the model with an example of the state progression to a potential failure state and the associated action necessary to correct it.

### 4.3 Results

**FIGURE 4:**
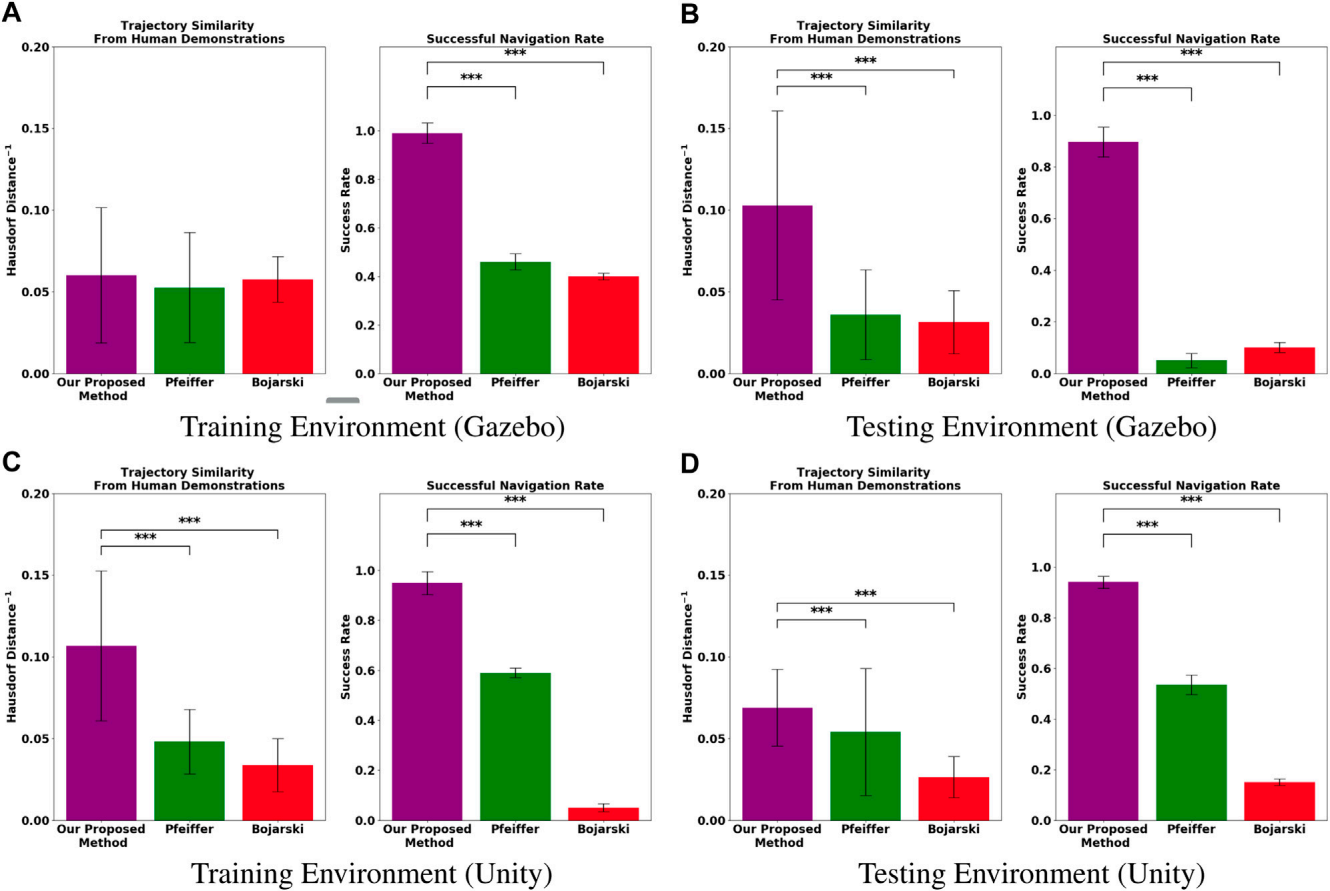
Experimental results evaluating the performances of each model trained using the baseline methods, Bojarski et al. (2016) (red) and Pfeiffer et al. (2018) (green), and our proposed method (violet). The top row shows results of new tasks performed within a **(A)** training environment, and tasks performed in a different **(B)** testing environment, both of which were simulated in Gazebo. The bottom row shows experiments conducted in **(C)** training and **(D)** testing environments simulated in Unity. The bar plot titled “Trajectory Similarity from Human Demonstrations” (left) uses Modified Hausdorff distance to quantify how similar the paths resulting from the policy rollouts are to an average of all human demonstrated paths for the same task. The bar plot titled “Successful Navigation Rate” (right) depict their success rates. The error bars represent the standard error computed over 50 trials. “***” indicates statistical significance, as computed using a Bonferroni corrected *p*-value of 0.025.

The results of our experiments are shown in Figure 4. They indicate that, for the fixed amount of demonstration data collected in our experiments, the proposed method can generate trajectories more similar to the ground truth human demonstrations with significantly higher completion rates than the models trained with the comparison methods. First, for each evaluation task, we recorded the success rate, i.e., the percentage of time that the system successfully navigated from the start point to the goal point. As seen in the subfigures labeled “Success Rate” in Figures 4A–D, the proposed method maintains a high task success rate in all four evaluation environments while the methods compared against do not. The second way in which we measured performance was trajectory similarity to the demonstrator. Note that, for evaluation, we compared the trajectories generated by each model to the human demonstrations of the evaluation task that were unique to the demonstrated trajectories used to train the model. The subfigures labeled “Hausdorff Distance^−1^” in Figures 4A–D report the inverse modified Hausdorff distance between the successful generated trajectories and the average human demonstration (i.e higher values indicate a greater similarity to the human demonstrations).

Additionally, we performed t-tests to determine statistical significance between our proposed method and our experimental baselines using a Bonferroni-corrected *p*-value threshold of 0.025. We observed that all experimental trials except those conducted in the Gazebo training environment (Figure 4A) were statistically significant. We posit that this is likely due the Gazebo training environment being the simplest environment among the four, making it easier for the all models to match the desired behavior demonstrated by the human user. The proposed method outperforms the competitors in this metric, meaning that its generated trajectories were more similar to those driven by the human.

Taken all together, our results demonstrate not only our method’s ability to learn navigation behavior like that which was demonstrated, but also its ability to generalize well beyond the training environment while retaining a high success rate.

## 5 Discussion

Our experimental results demonstrate the promise of the proposed method for performing Imitation Learning for autonomous robotic navigation. With very little human demonstration, our method was able to learn navigation behaviors like those demonstrated and retain a very high success rate. We posit that our technique was able to outperform others because it is able to appropriately leverage much of the existing machinery of autonomous navigation.

During our experiments, we noted that, while the methods from Bojarski et al. and Pfeiffer et al. were able to learn to navigate around each environment, they had a lower success rate than our approach. For the system proposed by Bojarski et al., which was designed for robust lane following, we observed the agent aimlessly navigating around the environment, avoiding the semantically labeled obstacles but never reaching the goal until the maximum number of time steps is reached. The method proposed by Pfeiffer et al. which does include a notion of global destination, did yield a higher success rate, though, again, not as high as the method proposed here.

While the results we presented do indeed establish the efficacy of our approach relative to the baselines, we also found that it has certain limitations. First, because our method relies on predicting discrete waypoints at a fixed distance, it is limited in the complexity of motion it can execute. While predicting waypoints to send to the local planner, saves us the hours of demonstration data needed to train a navigation policy as robust as an off-the-shelf navigation software, it does restrict the robot from performing highly precise maneuvers that models trained to predict wheel velocity and steering commands directly may be able to capture. Next, we noted that the proposed technique would sometimes generate waypoints within the inflation bounds of obstacles. When this happened, the local planner would still attempt to reach these invalid waypoints, resulting in task failure due to obstacle collision. In fact, the majority of unsuccessful navigation attempts recorded for the proposed system during our experiments were a result of this problem. This issue could possibly be mitigated through the use of explicit action-selection heuristics.

## 6 Summary

In this paper, we presented a novel method by which robotic agents can adapt their navigation behaviors in response to a demonstration of desirable behavior from a human user. Specifically, the proposed framework involved augmenting a traditional layered navigation system with a new machine learning module that performed BC. By training this module using the proposed procedure, we showed experimentally that the system can learn to imitate stylistic navigation behaviors while retaining the ability to perform successful navigation, even in an unseen environment.

## Data Availability

The raw data supporting the conclusions of this article will be made available by the authors, without undue reservation.
