# Supplementary figures and images for: Improving Autonomous Robotic Navigation Using Imitation Learning
Source: Front Robot AI. 2021 Jun 1;8:627730. doi: 10.3389/frobt.2021.627730 (PMC8204187; doi:10.3389/frobt.2021.627730)

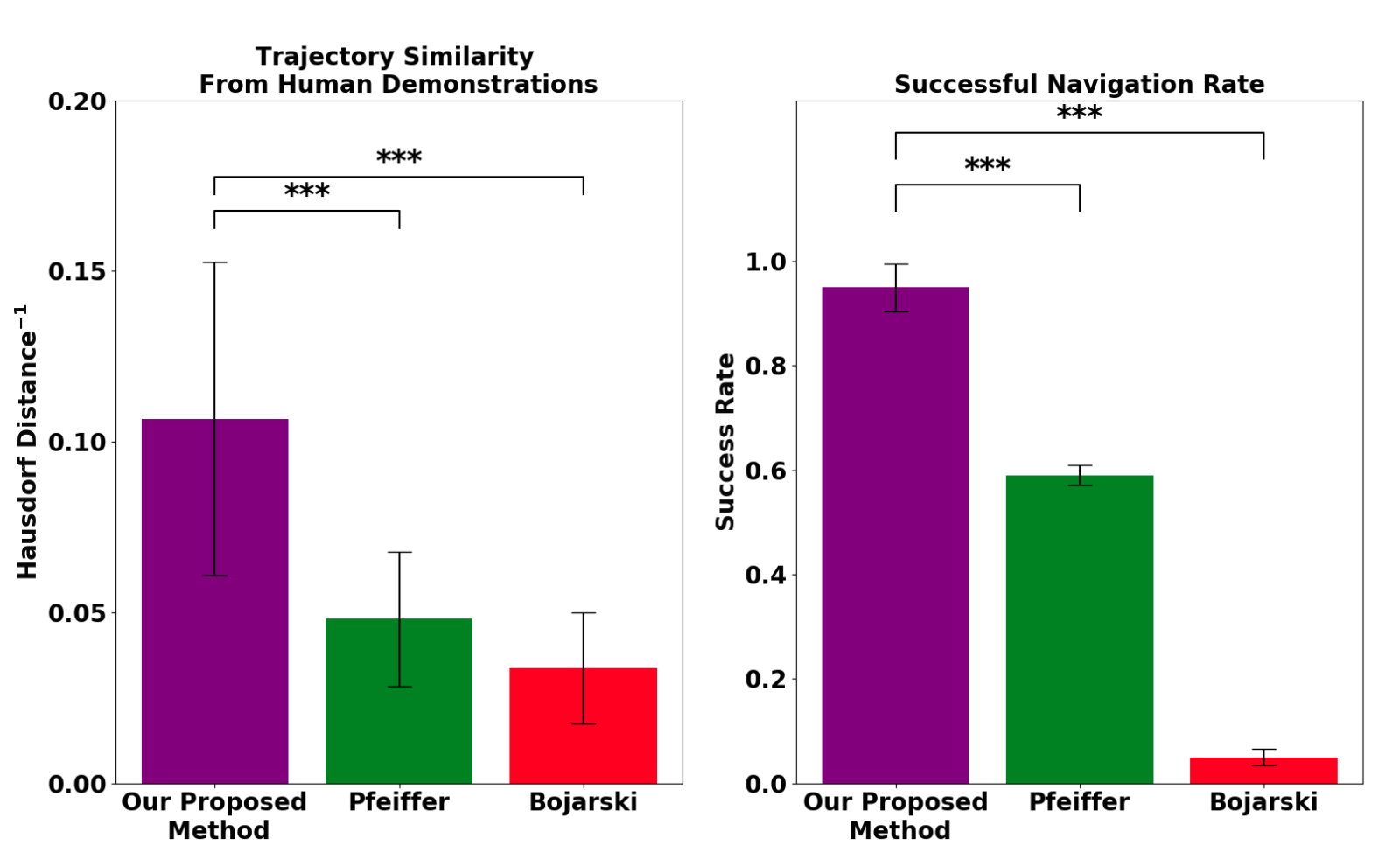

Supplement: Supplementary file 1 [file Image3.jpeg]

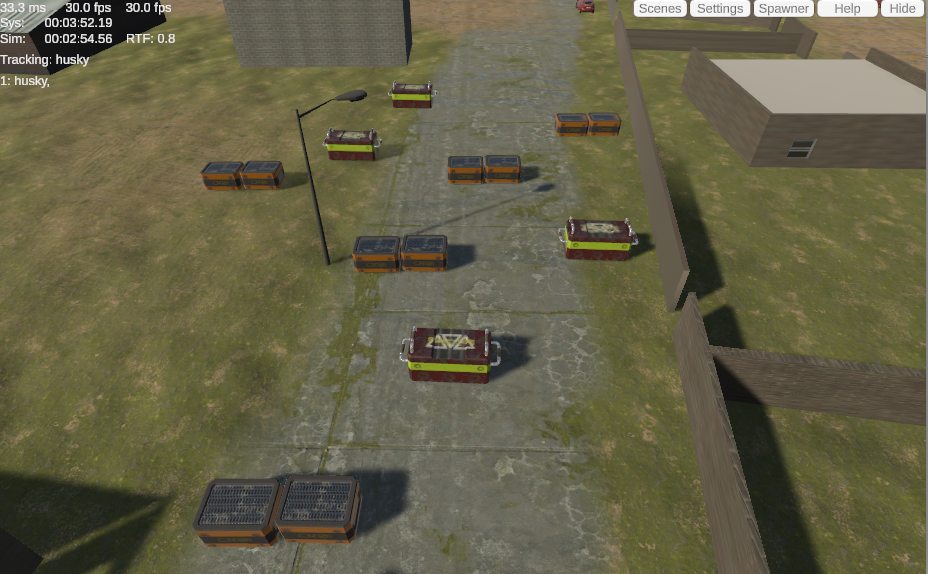

Supplement: Supplementary file 2 [file Image1.JPEG]

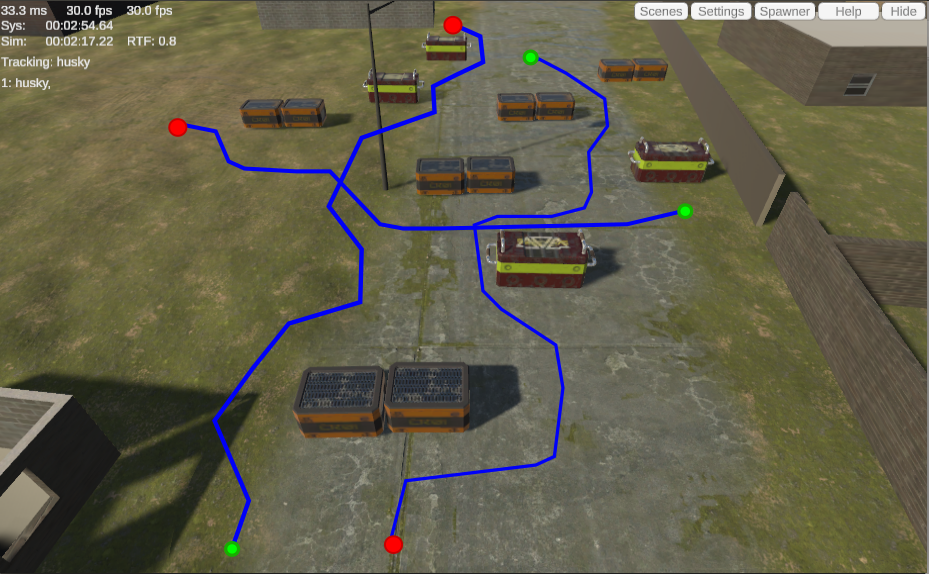

Supplement: Supplementary file 3 [file Image4.jpeg]

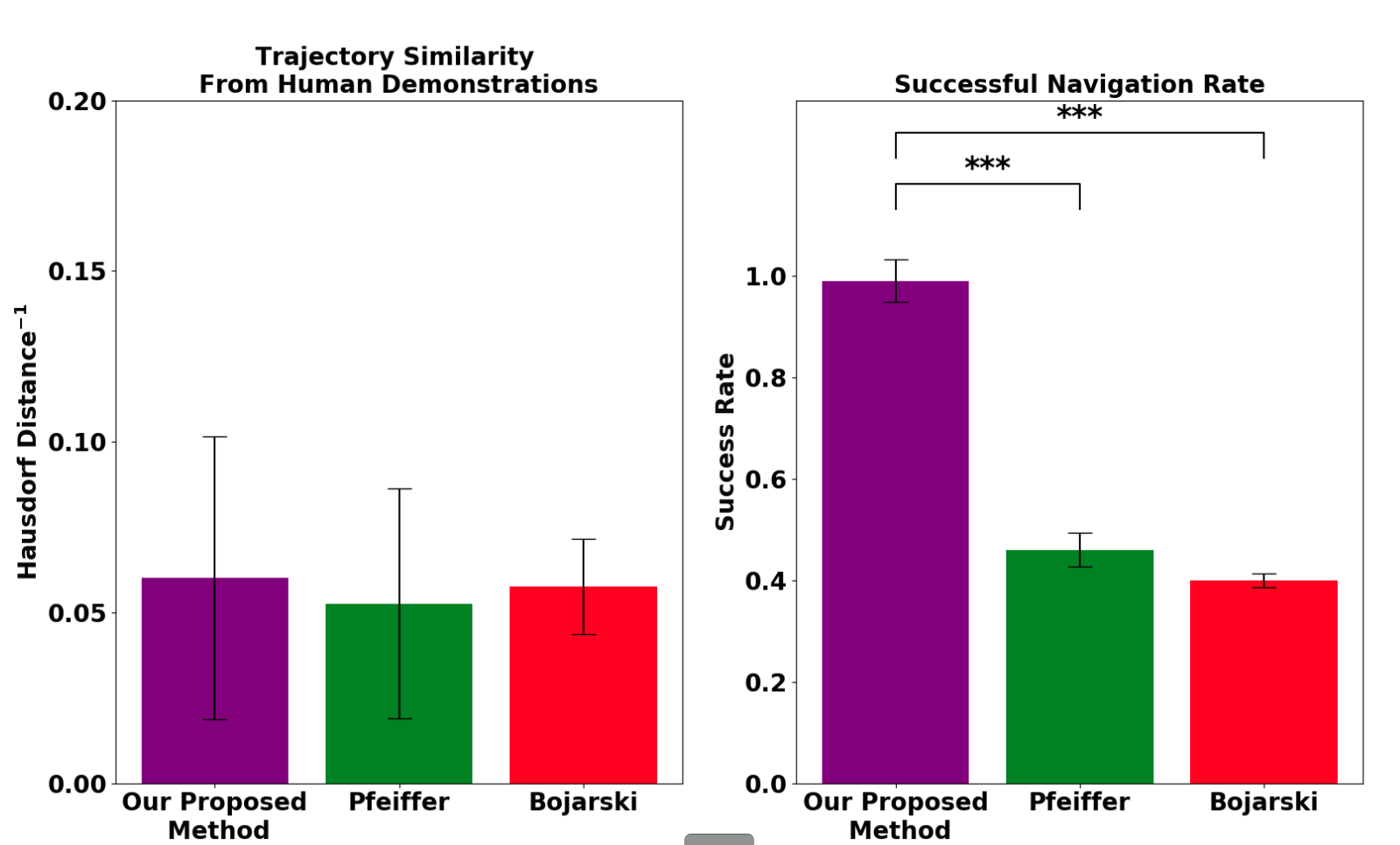

Supplement: Supplementary file 4 [file Image2.jpeg]

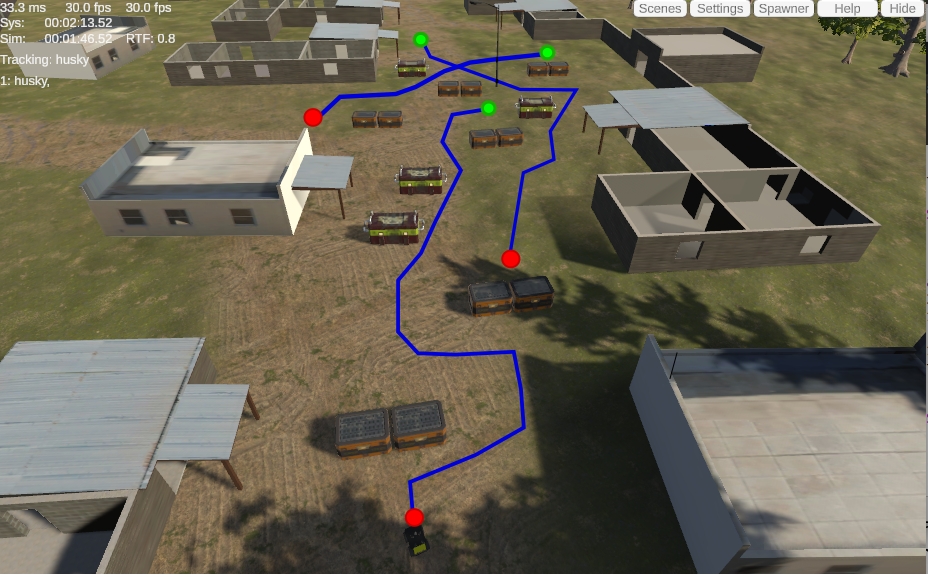

Supplement: Supplementary file 5 [file Image5.jpeg]
